# Non-stationary neural signal to image conversion framework for image-based deep learning algorithms

**DOI:** 10.3389/fninf.2023.1081160

**Published:** 2023-03-24

**Authors:** Sahaj Anilbhai Patel, Abidin Yildirim

**Affiliations:** Department of Electrical and Computer Engineering, The University of Alabama at Birmingham, Birmingham, AL, United States

**Keywords:** biomedical signals, non-stationary signal to 2D image representation, 2D convolution neural network (2D CNN), Bresenham’s line algorithm, electroencephalogram (EEG)

## Abstract

This paper presents a time-efficient preprocessing framework that converts any given 1D physiological signal recordings into a 2D image representation for training image-based deep learning models. The non-stationary signal is rasterized into the 2D image using Bresenham’s line algorithm with time complexity O(n). The robustness of the proposed approach is evaluated based on two publicly available datasets. This study classified three different neural spikes (multi-class) and EEG epileptic seizure and non-seizure (binary class) based on shapes using a modified 2D Convolution Neural Network (2D CNN). The multi-class dataset consists of artificially simulated neural recordings with different Signal-to-Noise Ratios (SNR). The 2D CNN architecture showed significant performance for all individual SNRs scores with (SNR/ACC): 0.5/99.69, 0.75/99.69, 1.0/99.49, 1.25/98.85, 1.5/97.43, 1.75/95.20 and 2.0/91.98. Additionally, the binary class dataset also achieved 97.52% accuracy by outperforming several others proposed algorithms. Likewise, this approach could be employed on other biomedical signals such as Electrocardiograph (EKG) and Electromyography (EMG).

## 1. Introduction

The Brian-Machine Interface (BMI) domain is a rapidly evolving multidisciplinary field that aims to bridge the neuron’s electrical response from extracellular or intracellular electrode arrays to man-made devices, commonly referred to as prosthetic devices (Carmena et al., 2003). The neural activities in different brain regions correlate to their conclusive motor control functions. For BMI, it is essential to decode neural signals accurately and classify them based on specific features. This would help to localize a specific type of neuron population in the brain, which is critical for implantable devices (Lebedev et al., 2011).

Since recording in the 1929s (Adrian and Bronk, 1929), many different methods have been developed to classify neural signals. Simple threshold methods have been used to detect the action potentials (Gerstein and Clark, 1964). Despite the simplicity, this method is efficient only with a high SNR. With advanced computerization and programming tools, various computational algorithms have been developed to classify efficiently and accurately the different types of neural activities (Lewicki, 1998). The primary two criteria for spike sorting or “clustering” consists of three basic steps: Filtering the signal, Feature representation or extraction, and Classification. Selecting an optimum preprocessing method with less computation time would inevitably help to perform the signal classification in real-time, which is one of BMI’s fundamental requirements.

In the preprocessing feature extraction or representation step, most researchers employed different Frequency and Time-Frequency (TF) domain methods that convert 1D non-stationary signals into a 2D image representation. These 2D images represent the different properties of a given neural recording signal. The Fast Fourier Transform (FFT) and Short-Time Fourier Transform (STFT) algorithms are commonly used frequency-based approaches for feature representation from neural recording (Boashash and Sucic, 2003). However, the FFT can only represent spectral resolution, and it is highly computationally intense compared to other TF methods with a time complexity of O(N log N) (Peng, 2020). The Short-Time Fourier Transform (STFT) overcomes the problem of the FFT method by adding temporal and spectral resolution (Mandhouj et al., 2021). Chaudhary et al. (2019) presented the Continues-Wavelet method that outperforms the STFT for classifying different Electroencephalogram (EEG) signal classes. However, selecting the mother wavelet according to the classification application is crucial while applying the wavelet method. In addition, the scale parameter of the mother wavelet set by the user determines the signal’s low or high temporal resolution.

Besides various TF representation methods (Brynolfsson and Sandsten, 2017; Khan et al., 2022), Anwar et al. proposed a new approach by placing multiple 2D EEG topographic maps of skulls into a single image from a given 1D segmented EEG signal (Anwar and Eldeib, 2020). Bizopoulos et al. (2019) proposed a “Signal-to-Image Module” that converts raw 1D signal samples into 2D Spectrogram images following up with one layer Convolution Neural Network (CNN) (Bizopoulos et al., 2019). However, the “Signal-to-Image Module” performance was inefficient without the CNN layer. Furthermore, adding one CNN layer increases computational time to O((M*N)*k2), where “M” and “N” represents image size while “k” represents kernel size. Zhao et al. (2021) proposed a Signal-To-Image Mapping (STIM) technique where the 2D image is formed by calculating the correlation between each sample point in the time series. The correlated sample points represent the grayscale by normalizing the data between 0 – 255. However, the converted 2D image does not preserve the signal space characteristics. Kavasidis et al. (2017) proposed the “*Brain2Image*” methodology using a deep learning model. The *Brain2Images* uses Long Short Term Memory (LSTM) autoencoder for converting multi-channel EEG signals into an image for 2D CNN model classification.

For systems with dynamic behaviors, many different methodologies are also proposed to analyze the behaviors of non-stationary signals in multi-dimensional space, such as Lyapunov exponents and fractal dimensions (Donner and Barbosa, 2008). Generally, such dynamical systems are utilized for analyzing the chaotic systems in low dimensions by reconstructing the phase–space (Packard et al., 1980). Such phase spaces are mapped into one dimension by extracting the positive characteristic exponent of the attractor. Over time, many scholars have developed different approaches to integrating the time series of dynamic systems with field graph theory (Takens, 1981; Yang and Yang, 2008). In graph theory, various methods have been developed to convert time series into graph networks, such as visibility graphs (represents condition on the time series amplitudes) (Lacasa et al., 2008). Later, the graph network is converted into a 2D image (such as Adjacency, Degree, and Laplacian matrix) representing the whole graph’s features and the time series non-linear properties (Donner et al., 2010). For example, Marwan et al. (2009) represented a complex network into a 2D recurrence matrix with its logistic map, where the recurrence matrix represents the neighbors in phase space. These 2D log-mapped recurrence matrices measured significant sensitivity to change in the dynamics. Shimada et al. (Myers et al., 2019) proposed a k-nearest neighbors approach in their 2D phase space matrix that represents fixed k numbers of single observations in given environments. The single observation can be fixed-phase space distance (constant volume, density). However, such a matrix does not preserve temporal information of given time series.

The conversion of 1D vector signals to 2D images is performed by implementing various “Signal Processing” methodologies such as FFT, Spectrogram, Empirical Mode Decomposition, and Wavelet. These methodologies and some others are time intensive and require high computational power. However, in computer graphics applications, many algorithms for image formation do not require high computing power. The computer graphics domain comprises four significant areas: Image Rendering, Modeling, Animation, and Postprocessing (Wolfe et al., 2000). For instance, in computer graphics, to render a line in 1D or 2D space (bitmap), two distinct pixel locations are selected, and the pixel locations between them are approximated. The Digital Differential Analyzer (DDA), a digital integrating computer (Bartee et al., 1962), is a line drawing algorithm. The DDA draws the lines that utilize the slope equation and approximates the next pixel value based on previous results. However, DDA only works in the first cartesian quadrant, and operating with floating-point numbers will increase the computational time. To overcome DDA, Bresenham (1965) proposed a method that approximates n-points in any given quadrant by calculating simple arithmetic integer operations such as addition, subtraction, and bit-shifting, that decreases computational time by O(n) where n represents the number of samples.

Generally, after converting the non-stationary signal into 2D image representation in BMI applications, the images or extracted features are directly fed into the classifying model to identify the different classes. In feature extraction, the most dominant features are extracted from multi-dimensional 2D images, which leads to reducing the input image size for classifying. For instance, the machine learning domain’s most common feature extraction technique is Principal Component Analysis (PCA) (Jolliffe, 2002). In PCA, the user selects the first few dominant features (known as Principal Components) for classification. After the state-of-the-art algorithm by Hilton. et.al (Krizhevsky et al., 2017), the CNN Deep Learning techniques are more commonly used for image classification tasks. They have been implemented in various applications such as Speech recognition, Self-Driving car, and classifying biomedical signals. Additionally, in recent years, CNN architecture has been improved by methods such as GoogleNet (Szegedy et al., 2015), Highway Network (Srivastava et al., 2015), VGG (Simonyan and Zisserman, 2014), and Xception (Chollet, 2017).

**FIGURE 1:**
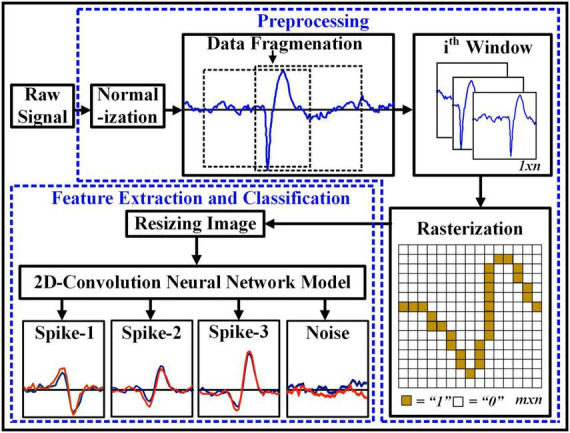
Overall block diagram of the proposed framework for neural spikes classification.

This paper presents a robust preprocessing approach to classify neural spikes in 2D CNN by converting 1D non-stationary neural recording into a 2D image representation. Figure 1 demonstrates the complete process of the proposed method. First, the simulated neural recordings are normalized between 0 and 1. Next, the entire recording is divided into smaller segments the size of 1 × 56. Then, the 1D segmented windows are converted into a 2D image by assigning each sample amplitude and interconnecting the sample points using Bresenham’s line algorithm (Bresenham, 1965). Finally, the 2D images are fed into the 2D CNN model for classification. The following sections of the paper are as follows: Section 2 presents the dataset. Section 3 explains the overall methodology, followed by three subsections – Signal Pre-processing, Rasterization, and Feature Extraction and Classification. The experimental result and discussion are described in section 4. Finally, section 5 concludes this paper.

## 2. Dataset

### 2.1. Dataset – 1

The dataset-1 consists of simulated action potentials with added noise (Bernert and Yvert, 2019). The timing information of the action potentials was also given. The data was generated by using equation (Eq.) 1 (Adamos et al., 2008).


(1) $$V\,\left(t\right)=\mathrm{Acos}\,\left(2\,\pi \,\frac{t-\tau_{\mathrm{ph}}}{\tau_{1}}\right)\,\exp\,\left(-{\left(\frac{2.3548\,t}{\tau_{2}}\right)}^{2}\right)$$

The dataset contains three types of action potentials with seven different SNRs ranging from 0.5 to 2. The parameters (*A*,τ_1_,τ_2_,τ_*ph*_) for generating three action potentials (spikes) are presented in Bernert and Yvert (2019). Each SNR trial contains ten recordings with three types of spikes, which are randomly distributed over a period of 200 s. The signal is stimulated at a sampling frequency (fs) of 20 kHz with a mean firing rate of 3.3 Hz. Table 1 illustrates the number of segmented windows for each class with different SNR levels. Note that each window is made of 56 samples. The number of windows (Table 1) for each spike type was configured to be nearly the same to prevent the deep learning model (2D CNN) from being biased toward classes with more segmented windows while training and testing the model.

**TABLE 1:** Number of windows for each class per SNRs in dataset 1.

SNR	Spike-1	Spike-2	Spike-3	Noise
0.5	6,423	6,595	6,597	7,759
0.75	6,444	6,591	6,597	7,759
1.0	6,419	6,591	6,597	7,761
1.25	6,394	6,587	6,597	7,758
1.5	6,279	6,585	6,596	6,500
1.75	5,987	6,574	6,597	7,756
2.0	5,553	6,633	6,597	7,750

### 2.2. Dataset – 2

Dataset-2 was collected from the University of California at Irvine (UCI) - Machine Learning Repository, which is utilized for Epileptic Seizure Recognition. The original dataset consists of brain activity from five subjects with five sets (100 channels/set) and 23.6 seconds per recording (Andrzejak et al., 2001). The dataset on UCI was already pre-processed with 178 samples per window and five classes to classify. In this paper, the dataset was converted from a multiclass category to binary classification (Seizure, Non-Seizure activity). The total number of windows and the number of classifying categories is presented in Table 2.

**TABLE 2:** Number of windows for each class in dataset 2.

Non-seizure activity	Seizure activity
9,200	2,300

## 3. Proposed methodology

### 3.1. Signal normalization and segmentation

Initially, each sample value of the raw signal is scaled by the unit L2 norm (ranging between 0 and 1) (Bhatia, 2013). The vector normalization of the raw signal was performed by using,


(2) $$y=\frac{x}{\sqrt{\sum_{i=1}^{d}x_{i}^{2}}}$$

where x is 1-D input samples values in a vector, y is 1-D normalized output in vector, and its L2 norm is$\sqrt{\sum_{\text{i}=1}^{\text{d}}{\text{x}}_{\text{i}}^{2}}$. Later, the normalized input vector is segmented into fixed 1Xn window sizes. Each window carries 56 sample values which are equal to 2.8 milliseconds.

### 3.2. Rasterization

Rasterization is a process of converting any vector graphic shape into a pixel image (Worboys and Duckham, 2004). For instance, Bresenham’s line algorithm approximates n-points between given two points of interest or locations of interest in any coordinates of Euclidean space.

The prediction for the next n*^th^* point is determined by calculating the least distances, i.e., q and t, which are perpendicular to the slope line - m of given two-pixel positions.

**FIGURE 2:**
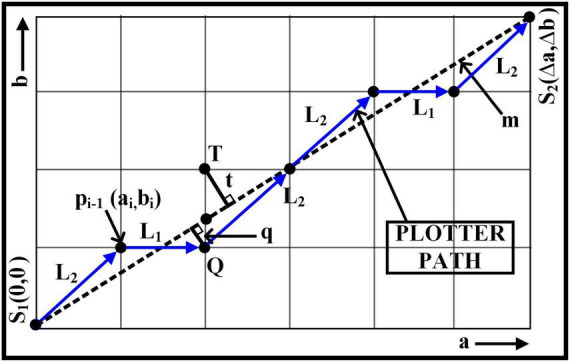
Bresenham’s line algorithm for first octant.

For instance, in Figure 2, points S_1_ and S_2_ are placed adjutant to each other in the first octant. Therefore, to predict the next decision parameter for the point, the movement can be either L_1_ (to point Q) if *q* < t or L_2_ (to the point *T*) if *t*≥q*respectively*. Therefore, the difference between q and t is calculated to compute the next move. If we define the difference as: ∇*_i_* = *q*−*t*, then;


$$\mathrm{if}\,\{\begin{array}{c} \nabla_{i}<0,e\,x\,e\,c\,u\,t\,e\,L\,1\\ \nabla_{i}\geq 0,e\,x\,e\,c\,u\,t\,e\,L\,2 \end{array}$$

The initial point S_1_ where *a=0* and *b=0* the ∇_*i*_ will as expressed in equation 3, and the points p_*i* + 1_ which is progressing toward S_2_ after the initial stage is calculated based on ∇_*i+1*_ which is Eq. 4 and Eq. 5.


(3) $$\nabla_{i}=2\,\Delta \,b-\Delta \,a$$

If ∇*_i_*≥0,


(4) $$\nabla_{i+1}=\nabla_{i}+2\,\Delta \,b-2\,\Delta \,a$$

If ∇*_i_* < 0,


(5) $$\nabla_{i+1}=\nabla_{i}+2\,\Delta \,b$$

where,


$$\Delta \,a=x_{2}-x_{1}$$

$$\Delta \,b=y_{2}-y_{1}$$

**FIGURE 3:**
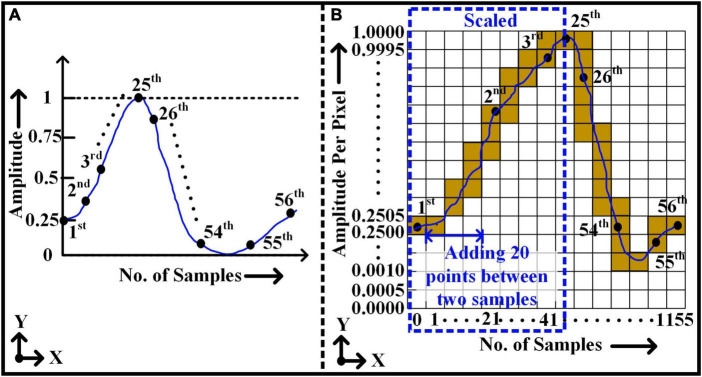
Rasterization approach - converting 1D vector of non-stationary signal to 2D pixel image. **(A)** 1D window frame – 1 × 56 size. **(B)** 2D window frame after rasterization - 2001 × 1155 size.

Figure 3A illustrates the normalized window with a total of 56 sample points (i.e., *y*-axis) over time (i.e., *x*-axis). Figure 3B shows the conversion of (1 × 56) window size to an image (2,001 pixel × 1,155 pixel) format applying Bresenham’s line algorithm. Each pixel in the direction of the y-axis of the image represents the amplitude value of the action potential with a resolution of 0.0005. Likewise, the column pixels in the x-axis direction represent the sample locations. Initially, all samples of 1D window representation are placed in their appropriate locations in 2D images as a pixel value of 1 or 0 accordingly. As shown in Figure 3B, to reconstruct a continuous uniformity pattern for the signal, two consequent real sample locations are filled with the dummy pixels. For instance, it is assumed that each window includes 20 empty dummy samples over an interval between each two real sample locations. The assumption of dummy samples resulting a satisfactory representation of the signal curve by connecting the points with line segments. However, the length of intermediates samples can be varied to reduce the overall size of the image. Later, using Bresenham’s line algorithm, all true sample values are interconnected, and it represents a complete shape or pattern of the signal in a 2D image.

### 3.3. Feature extraction and classification

**FIGURE 4:**
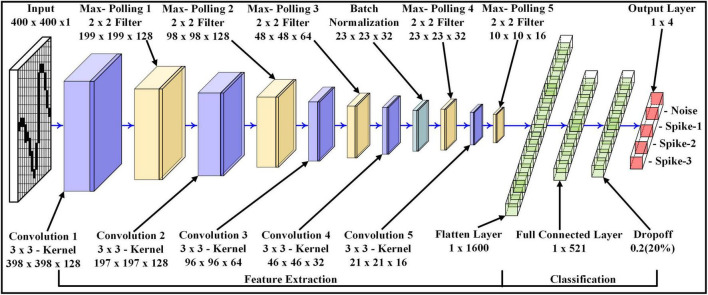
Modified 2D CNN architecture for feature extraction and classification of four classes.

The 2D CNN architecture is used to extract the features (in the spatial domain) of the signals and then classify them according to different signal types and different SNRs. The proposed approach was performed on custom-built 2D CNN architecture [extended LeNet-5 architecture (LeCun et al., 1998)], as shown in Figure 4. The 2D CNN model consists of five different sizes of convolution layers and five max-pooling layers. Each convolution layer extracts/preserves different spatial features, which are also called feature maps. These feature maps are obtained by convoluting the input (output of the previous layer) with a 3×3 kernel size (Eq. 6) followed by a non-linear Rectified Linear Activation Unit (ReLU) activation function (Eq. 7). The non-linear activation function extracts more complex features. The output of each convolution layer is connected to the max-pooling layer. The purpose of the max-pooling layers is to extract maximum spatial values from each feature map. In addition, these reduce the convolution layer size by half, which leads to a decrease in the number of training weights parameters for the 2D CNN model. The output size of each feature map and pooling layer can be calculated by Eq. 8 and Eq. 9 accordingly.


(6) $${\mathrm{COV}}_{\mathrm{ij}}^{L}=\sum_{a=0}^{k-1}\sum_{b=0}^{k-1}\omega_{\mathrm{ab}}\,{\mathrm{COV}}_{\left(i+a\right)\,\left(j+b\right)}^{L-1}$$

where,

$C\,O\,V_{i\,j}^{L}$ = Output per pixel in convolution layer, ω_*ab*_ = weights per pixel in convolution layer, *k* = Kernel size, *L* = Convolution layer.


(7) $$Y_{\mathrm{ij}}^{L}=f\,\left({\mathrm{COV}}_{\mathrm{ij}}^{L}\right)$$

where,

*f* = ReLU or SoftMax activation function,


(8) $${\mathrm{COV}}_{\mathrm{out}}=\left[\frac{N_{c}+2\,P_{c}+K_{c}}{S_{c}}\right]+1$$

where,

*COV*_*out*_ = Feature map output size, *N*_*c*_ = Input size (Width/Height), *P*_*c*_ = Convolution Padding size, *K*_*c*_ = Convolution Kernel size and *S*_*c*_ = Convolution Stride size.


(9) $$P_{\mathrm{out}}=\left[\frac{N_{p}-K_{p}}{S_{p}}\right]+1$$

where,

*P*_*out*_ = Pooling layer output size, *N*_*p*_ = Input size (Width/Height), *K*_*p*_ = Pooling Kernel size, *S*_*c*_ = Pooling Stride size.

In the initial stage of convolution layers, the number of feature maps is higher than in the end convolution layers. For instance, Convolution 1 has 128 feature maps, while Convolution 5 has only 16 feature maps. Therefore, the arrangement of such layer size was used to extract larger patterns compared to finer patterns. The batch normalization is placed between the fourth convolution layer and the fourth max-pooling layer. It potentially helps to converge loss values of the deep learning model much faster in mini-batches, which contain small bundles of data. This leads to a reduction in time consumption during the training phase of the deep learning model.

After the fifth max-pooling layer, the 3D tensor feature maps are converted to 1×1600 1D vector space, called a flattened layer. The next layer with the size of 1×512 is called the Fully Connected Layer (FCL), where each artificial neuron in the FCL layer is interconnected with the artificial neurons of the previous or next layer. The following layer is called the Dropout Layer (DL), which is connected to the FCL and has the same vector size as FCL. The DL functions as regularization, which prevents the 2D CNN model from over-fitting the training dataset while training the weight parameters. In DL, the random neurons in the layer and their supportive links are eliminated.

Finally, the DL is connected with the Output Layer with the size of 1×4 for Dataset-1 and 1×2 for Dataset – 2. The vector size of the output layer represents the number of classes to be identified by the 2D CNN model. For the output layer, the SoftMax activation function is deployed. The SoftMax activation function determines the outcome probabilities of each class.

The RMSprop optimizer (Tieleman and Hinton, 2012) is used while training the 2D CNN model to reduce classification loss error (*E*). This error rate can be reduced by backpropagation. In backpropagation, the weight parameters for each layer are updated by finding the partial derivative of *E* w.r.t individual weight parameter per layer that is $\left(\frac{\partial E}{\partial {\mathrm{COV}}_{\mathrm{ij}}^{L}}\right)$.

## 4. Results and discussion

The proposed framework was executed on the University of Alabama at Birmingham supercomputer. The supercomputer is configured with 24 Cores and 23 GB of memory per Core. The method was trained and evaluated using a 2D CNN model with various SNRs. Additionally, to demonstrate the robust performance of the proposed approach for other applications, the 2D CNN model was also trained and evaluated on the EEG dataset (Dataset-2). During each trial, the parameters for 2D CNN architecture are kept the same, such as the number of epochs, batch size, optimizer, input image size, and split ratio for training and testing datasets except output classes. The total number of windows for Dataset-1 and Dataset-2 is split into 30% training and 70% test dataset, respectively. Table 3 and Table 4 illustrate the total number of training and test sample windows for each SNR and dataset-2 accordingly.

**TABLE 3:** Number of training and testing dataset per SNRs in dataset 1.

SNR	Train	Test	Total
0.5	8,211	19,160	27,371
0.75	8,217	19,174	27,391
1.0	8,210	19,158	27,368
1.25	8,200	19,136	27,336
1.5	7,788	18,172	25,960
1.75	8,074	18,840	26,914
2.0	7,959	18,574	26,533

**TABLE 4:** Number of training and testing dataset for dataset 2.

	Train	Test
Normal Activity	2,744	6,456
Seizure Activity	706	1,594

**FIGURE 5:**
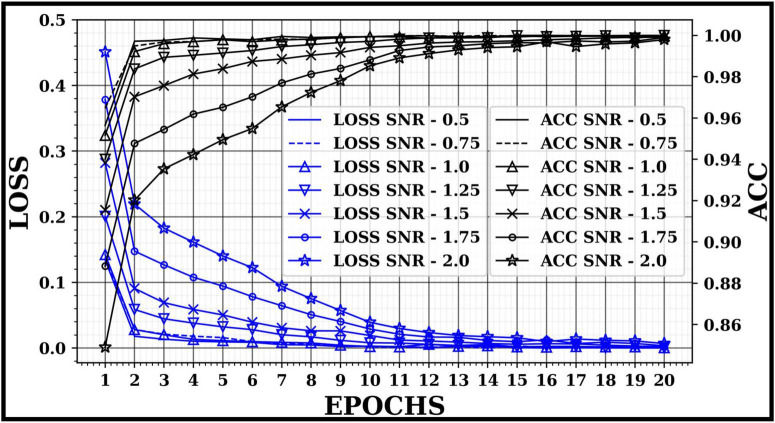
Loss vs. no. of epochs per SNR’s and accuracy vs. epochs.

Figure 5 demonstrates the accuracy (ACC) and loss of the 2D-CNN model for each SNR according to the number of epochs while training the training dataset. The optimizer was set with a 0.001 learning rate and 20 epochs. Figure 5 also shows that in images with higher SNR, the loss converges faster to a minimum in a few epochs, in contrast to images with low SNR. In this study, to verify the overall performance of the proposed approach on different images with different SNRs, the number of epochs is kept similar for all trials while training the 2D CNN model.

The data splitting approach was implemented for cross-validating the 2D CNN model. To prevent the 2D CNN model from being biased toward any of the classes, the input data was shuffled and divided almost equally for each class except for Dataset 2. The performance of the approach was calculated based on results predicted by a trained 2D CNN model on 70% of the test data by comparing its ground truth results. Tables 5, 6 represent the precision ratio, recall ratio, F1-score for each class, and overall ACC for each SNR (Dataset-1) and Dataset-2, respectively. The precision ratio, recall ratio, F1- score, and ACC are calculated using Eq. 10, Eq. 11, Eq. 12, and Eq. 13, respectively.


(10) $$\mathrm{Precision}=\frac{\mathrm{TP}}{\mathrm{TP}+\mathrm{FP}}*100$$

(11) $$\mathrm{Recall}=\frac{\mathrm{TP}}{\mathrm{TP}+\mathrm{FN}}*100$$

(12) $$F\,1-\mathrm{Score}=\frac{2*\left(\mathrm{Precision}*\mathrm{Recall}\right)}{\left(\mathrm{Precisioin}+\mathrm{Recall}\right)}*100$$

(13) $$\mathrm{Accurary}=\frac{\mathrm{TP}+\mathrm{TN}}{\mathrm{TP}+\mathrm{FP}+\mathrm{TN}+\mathrm{FN}}*100$$

**TABLE 5:** Performance of proposed approach with 2D CNN for different SNR signals – Precision ratio, recall ratio, F1-score and accuracy rate.

SNR	Class	Precision ratio (%)	Recall ratio (%)	F1-score (%)	Accuracy rate (%)
0.5	Noise	99.81	99.63	99.72	99.77
	Spike-1	99.73	99.80	99.76	
	Spike-2	99.69	99.89	99.79	
	Spike-3	99.84	99.80	99.82	
0.75	Noise	99.68	99.63	99.66	99.69
	Spike-1	99.75	99.84	99.80	
	Spike-2	99.52	99.67	99.59	
	Spike-3	99.80	99.63	99.71	
1.0	Noise	99.74	99.57	99.65	99.49
	Spike-1	99.79	99.84	99.82	
	Spike-2	99.60	98.79	99.19	
	Spike-3	98.83	99.76	99.29	
1.25	Noise	99.79	98.64	99.21	98.85
	Spike-1	98.94	99.88	99.41	
	Spike-2	98.20	98.20	98.20	
	Spike-3	98.31	98.77	98.54	
1.5	Noise	98.83	97.90	98.36	97.43
	Spike-1	99.06	99.61	99.21	
	Spike-2	96.55	94.71	95.63	
	Spike-3	95.38	97.84	96.59	
1.75	Noise	95.11	98.15	96.61	95.20
	Spike-1	98.89	96.63	97.75	
	Spike-2	92.35	92.07	92.21	
	Spike-3	94.90	93.53	94.53	
2.0	Noise	94.61	94.03	94.32	91.98
	Spike-1	95.78	96.36	96.07	
	Spike-2	90.21	83.73	86.85	
	Spike-3	87.64	94.12	90.76	

**TABLE 6:** Performance of proposed approach with 2D CNN for dataset 2 – Precision ratio, recall ratio, F1-score and accuracy rate.

Class	Precision ratio (%)	Recall ratio (%)	F1-score (%)	Accuracy rate (%)
Seizure activity	98.33	98.59	98.46	97.52
Normal activity	94.22	93.22	93.73

where,

TP = True Positive, FP = False Positive, TN = True Negative, FN = False Negative.

**FIGURE 6:**
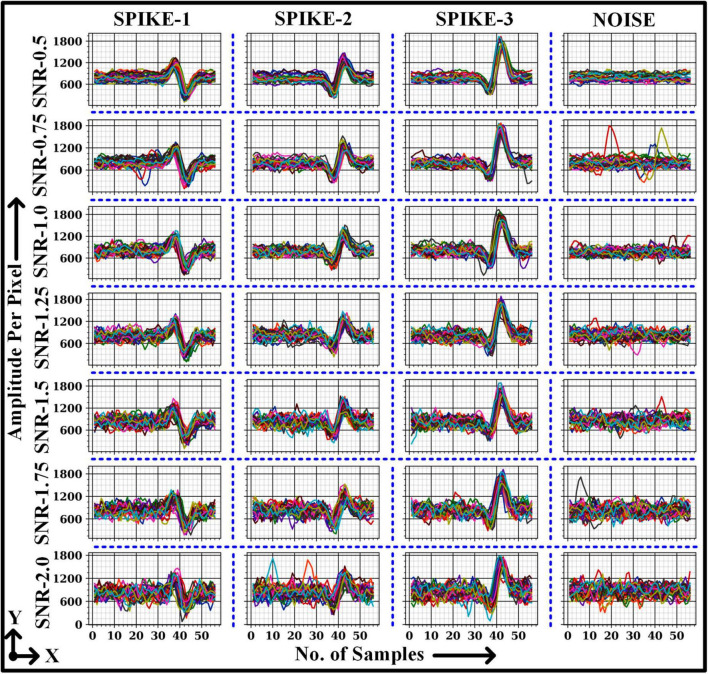
2D CNN model predicted output on test dataset for various images with SNR signals.

The overall ACC for images with signals 0.5, 0.75, 1.0, 1.25, 1.5, 1.75, and 2.0 SNR was above 90%. Despite this, the average ACC for 2.0 SNR was lower than other SNRs because of the higher number of False Positive detection for class “Spike-2”. Figure 6 illustrates a few classified results of the testing dataset with images having different SNRs. It also demonstrates a few misclassified outputs in the “Noise” class at “SNR 0.75.” The overall ACC for images in Dataset-2 was 97.52%. The performance of this study was compared with other studies in related fields based on Dataset-2, as shown in Table 7.

**TABLE 7:** Comparison of proposed approach with other studies.

Studies	ACC
K-nearest neighbor (Resque et al., 2019)	90.01
Multi-layer perceptron (Resque et al., 2019)	93.53
Naive-Bayes (Resque et al., 2019)	95.98
Random forest (Resque et al., 2019)	97.08
1D CNN (Xu et al., 2020)	97.13
Support vector machine (Resque et al., 2019)	97.31
Our method	**97.52**
CNN-LSTM (Xu et al., 2020)	99.39

## 5. Conclusion

This work demonstrated an efficient preprocessing framework to convert any non-stationary signal into a 2D image using Bresenham’s line algorithm. The 2D images can utilize any image classifying algorithm or image-based deep learning models. The experiment was conducted on neural data with various SNR and EEG recordings. The proposed approach showed empirical evidence of performance accuracy with various SNRs and fewer data samples for training. The overall classification accuracy for each SNR outcome (SNR/ACC) as: 0.5/99.69, 0.75/99.69, 1.0/99.49, 1.25/98.85, 1.5/97.43, 1.75/95.20 and 2.0/91.98. In addition, the 2D CNN also performed significantly well on the EEG dataset compared to other methods, with 97.52% ACC. The proposed pre-processing framework could be used in real-time because of the advantage of the low computation time of Bresenham’s line algorithm.

## Data availability statement

The original contributions presented in this study are included in the article/Supplementary material, further inquiries can be directed to the corresponding author.

## Author contributions

SP developed the framework for this research and drafted the manuscript. AY helped to revise the drafted manuscript and provided valuable advice for this research. Both authors read and approved the final manuscript.
