# Supplementary material for: Non-stationary neural signal to image conversion framework for image-based deep learning algorithms
Source: Front Neuroinform. 2023 Mar 24;17:1081160. doi: 10.3389/fninf.2023.1081160 (PMC10079945; doi:10.3389/fninf.2023.1081160)
Supplement: Supplementary file 1 [file Data_Sheet_1.DOCX]

Supplementary Material

# Supplementary Data

The dataset 1 analyzed for this study can be found in the GitHub repository “https://github.com/sahaj432/syntactic_neural_recording.git” .

The dataset 2 analyzed for this study can be found in the GitHub repository “https://github.com/sahaj432/Epileptic_Seizure_Recognition_dataset.git” .
